# The influence of social motivation diversity on group creativity: evidence from fNIRS hyperscanning

**DOI:** 10.3389/fpsyg.2025.1665583

**Published:** 2025-10-20

**Authors:** Ning Liu, Sai Nan Ren

**Affiliations:** ^1^School of Psychology, Hainan Normal University, Haikou, China; ^2^Hainan Provincial Research Base for Psychological Development and Education of Adolescents, Haikou, China; ^3^Key Laboratory of Data Science and Smart Education, Ministry of Education, Hainan Normal University, Haikou, China

**Keywords:** group creativity, social motivation, group diversity, social motivation diversity, fNIRS

## Abstract

Social motivation diversity, defined as the heterogeneity in group members’ preferences for maximizing either individual (pro-self) or collective (pro-social) outcomes, remains underexplored in its neural correlates with group creativity. This study employed functional near-infrared spectroscopy (fNIRS)-based hyperscanning to investigate interpersonal neural synchronization (IBS) during creative collaboration in 60 dyads (30 diverse, 30 homogeneous) of university students (*N* = 120), experimentally assigned based on social motivation. Each dyad completed both a creative task (umbrella design improvement) and a non-creative task (umbrella purchase plan). Results revealed that the diversity group showed significantly higher IBS increments in the prefrontal cortex (channels CH20 and CH23) compared to the non-diversity group. Furthermore, IBS in CH23 was positively correlated with novelty scores in the creative task. These findings suggest that social motivation diversity enhances group creativity through increased neural synchrony, supporting the notion that pro-self members contribute novel ideas while pro-social members facilitate integrative cooperation. This study provides novel neurophysiological evidence for the role of motivation-based diversity in collaborative creativity.

## 1 Introduction

Creativity has been extensively studied at both the individual and group levels, with distinct conceptualizations for each domain. Individual creativity is typically defined as the production of novel and useful ideas by a single person (Amabile, 1982). In contrast, group creativity represents a more complex phenomenon involving the integration of diverse perspectives, knowledge sharing, and collaborative problem-solving among team members (Paulus and Brown, 2007). This collective process often generates innovative outcomes that transcend what any individual member could produce alone, emerging from the dynamic interactions within the group (Woodman et al., 1993). The transition from individual to group creativity introduces unique social and cognitive processes that merit distinct theoretical and empirical consideration. Creativity can be divided into individual creativity and group creativity according to different creative subjects, group creativity, that is, individuals work together in a complex social system to create products, ideas, procedures and processes with both value, usefulness and novelty (Woodman et al., 1993), which is affected by group social motivation (Zhang et al., 2020), group diversity (Zhang et al., 2016) and other group-related environmental variables.

Group diversity can be defined as the distribution of individual attributes of group members (Liu and Xu, 2006; Zhang et al., 2016; Tasheva and Hillman, 2019), the impact of group diversity on group creativity has been concerned by the academic community, for example, group professional (or knowledge) heterogeneity (Paulus and Nijstad, 2004) is one of the earliest group diversity variables that researchers pay attention to, the higher the degree of knowledge heterogeneity of group members, the higher the level of group creativity (Lv and Zhang, 2015), and some studies have explored the effects of gender and age diversity on group creativity (Wang et al., 2019). In fact, group diversity is not only the diversity of age, gender, and knowledge and profession that can affect group creativity (Wang, 2023). Acar et al. (2018) pointed out that motivation is a key mechanism that influences group ideas and experiments, and will have an important impact on group innovation.

Most studies of group creativity have been premised on different members of the same group having similar social motivations (Goette et al., 2012). The same assumption that each member of a group has the same social motivation tendencies is implicit in the motivated information processing in groups model (De Dreu et al., 2008). Shi’s (2015) preliminary exploration of the influence of social motivation on group creativity found that the group creativity of a prosocial motivation group was significantly higher than that of an egoistic motivation group, and the ideas generated by the prosocial motivation group were significantly more novel. However, few studies have comprehensively considered the impact of diversity of social motivation on group creativity. Referring to Tasheva and Hillman’s (2019) definition of group diversity, this study defines the diversity of social motivation in a group as the degree to which members of the same group differ in social motivation.

In groups without a diversity of social motivations, the members might all have a tendency toward being motivated by self-interest or by prosociality. In the former case—when all members of the group are motivated by self-interest—each individual wants the other members to obey their wishes. Poor communication or even mutual disagreement is thus unavoidable. The result of poor communication among group members is a lack of smoothness in the sharing and exchange of information and emotions, which is not conducive to group innovation behavior and thereby reduces the group’s creativity (Yang and Zhang, 2012). In the latter case, when all members of the group are prosocially motivated, they might prioritize conflict-avoidance and maintaining the superficial harmony of the group over carefully examining and exploring the task information in depth or expressing or insisting on their own opinions. They might therefore adopt a simple majority-rules approach to group decision-making, which encourages superficial information processing and has a negative impact on group creativity (De Dreu et al., 2008). These findings indicate that a lack of social motivation diversity in a group has a negative impact on creativity, regardless of whether the group’s tendency is toward self-interested or prosocial motivation.

When the individual members of a group have different social motivations, however, it is unclear what role this social motivation diversity plays. Given the above findings, it is reasonable to suggest that in a group containing both self-interested and prosocially motivated people, those motivated by self-interest will be able to put forward and adhere to their own views while those who are prosocially motivated will be able to actively seek cooperation to achieve a win-win situation. Nevertheless, the effect of group social motivation diversity on group creativity remains unknown, as do any interpersonal neural correlates of these group social motivation diversity effects. Accordingly, the current study addresses two research questions: (1) how does social motivation diversity in a group affect the group’s creative outcomes? (2) What are the interpersonal neural correlates underlying any effect of group social motivation diversity on group creativity? Addressing these questions can identify group creativity effects of social motivation diversity, deepen the understanding of these effects by exploring interpersonal neural correlates, and inspire future research innovation in the field.

The hyperscanning technique is adopted in this study to explore the interpersonal neural correlates of interest. Hyperscanning involves the simultaneous recording of the neural responses of multiple interacting individuals in real time. Researchers have confirmed an association between higher IBS and group creativity (Lu et al., 2018; Mayseless et al., 2019; Xue et al., 2018). For the current study, fNIRS-based hyperscanning was selected because of its advantages of a higher tolerance for motion artifacts and greater ecological validity than EEG or fMRI approaches and for allowing verbal communication during the scanning process.

A group is defined by Forsyth (2014) as two or more individuals who are connected by social relationships. In this study, owing to the limited number of fNIRS detectors and emitters in a practical montage, we could record simultaneous neural responses in the brain regions of interest of only two people; therefore, the dyadic paradigm was adopted (Lu et al., 2018; Mayseless et al., 2019). The participants, who were unknown to each other, had their social motivations manipulated and were divided into 30 social motivation non-diversity pairs (both members having prosocial motivation or both members having egoistic motivation) and 30 social motivation diversity pairs (one member having prosocial motivation and the other member having egoistic motivation). Each pair was required to solve two problems: one demanding creativity (a design improvement design tasks for umbrellas) and one not demanding creativity (an umbrella purchase plan task). While the participants carried out these tasks, we used fNIRS-based hyperscanning to simultaneously scan the neural responses of the participants in each pair. Neuroimaging studies in the field of social interaction and creativity have shown a strong association between the prefrontal cortex and the right temporoparietal symphysis and social interaction and creative cognitive processes, and previous hyperscanning studies have found a certain link between brain synchronization and group creative activity in these regions (Xue et al., 2018; Lu et al., 2018). This method was used to test the following hypotheses.

*H1*: The group creativity of social motivation diversity groups is higher than that of non-diversity groups.

*H2*: The effect of socially motivated diversity on group creativity differs between creative and non-creative tasks.

*H3*: Social motivation diversity groups have superior creative communication outcomes and greater IBS increments than non-diversity groups.

## 2 Methods

### 2.1 Participants

One hundred and twenty full-time college students (104 females, 16 males) were recruited. All participants were drawn from freshmen to seniors across all academic fields. The participants were assigned to a diversity group or a non-diversity group and divided into 60 pairs. Among them, there was no diversity group (both subjects were prosocial motivated or both were altruistic motivated), and diversity group (one subject was prosocial motivated and one was altruistic motivated). Before conducting the experiment, we confirmed that the participants in each pair were unknown to one another. Informed consent was obtained from the participants, and each participant was paid 20 yuan for taking part. It should be noted that the majority of participants in this study were female, reflecting the gender distribution of the recruitment pool. Future studies should aim to recruit a more gender-balanced sample to examine the potential influence of gender on the observed effects.

### 2.2 Experimental procedure

**Figure 1:**
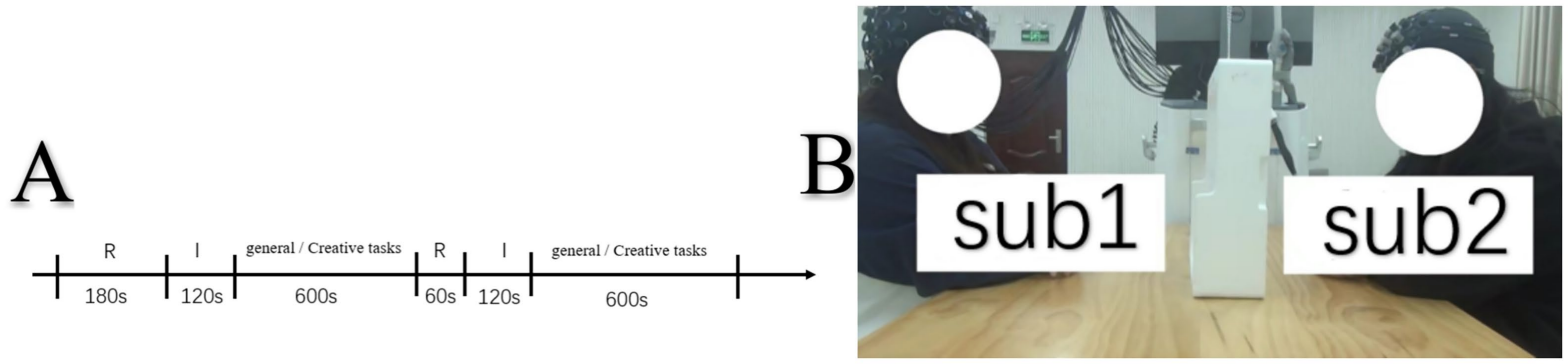
**(A)** Experimental design, R = resting state session; I = 120-s instruction session. **(B)** Experiment setting.

Upon arrival, each pair of participants sat face-to-face with two square tables between them (see Figure 1B). The experiment consisted of two 1-min resting sessions, two 2-min instruction sessions, and two 10-min task sessions (see Figure 1A). During the resting sessions, the participants were asked to close their eyes, remain still, and relax. During the instruction sessions, the task procedures were described in detail.

The creative task was adapted from the ‘Product Improvement’ subtest of the Torrance Tests of Creative Thinking (TTCT; Torrance, 1974) to facilitate group discussion. The originality, feasibility, and elaboration of the solutions were evaluated by independent raters using the Consensual Assessment Technique (Amabile, 1982), ensuring the validity of the creativity measurement. To provide more content for group discussion, we used an umbrella as the target everyday object to replace the toy elephant in the TTCT task. The groups were thus required to complete the “Umbrella Design Improvement” task. The non-creative task was to devise an umbrella purchase plan and was thus called the “Umbrella Purchase Plan” task. This task gave limited space for questions and did not require creative thinking (Li, 2021). The instructions and requirements provided for the experimental tasks were as follows:

*Prosocial motivation subject guidance*. “Your task in this experiment is to maximize the results of yourself and your team, evaluate the final results in team form, and receive a 40 yuan ‘Excellent Team Award’. Your role is confidential and you cannot discuss issues related to your role. You cannot write down your opinions without team discussion.”

*Egoistic motivation subject guidance*. “Your task in this experiment is to maximize your own achievements, evaluate the final results in personal form, and receive a 20 yuan ‘Personal Excellence Award’. Your role is confidential and you cannot discuss issues related to your role. You cannot write down your opinions without team discussion.”

*Umbrella Design Improvement (creative) task prompt*. “Umbrellas are indispensable at home! The following picture shows the most common type of umbrella. Please freely discuss and improve the creative design of the umbrella in the picture together, and finally form a complete set of creative design improvement plans!”

*Umbrella Purchase Plan (non-creative) task prompt*. “Umbrellas are indispensable at home! The following picture shows the most common type of umbrella. Please freely discuss together how to buy a satisfactory umbrella.”

The final result of the team form evaluation was awarded an ‘Excellent Team Award’ of 40 yuan to activate prosocial motivation, and the final result of the individual form evaluation was awarded a ‘Personal Excellence Award’ of 20 yuan to activate egoistic motivation. During the experiment, we told the participants that their roles were confidential and they were not to discuss issues related to their roles. To prevent teamwork from being abandoned, the participants were not allowed to write down ideas that had not been discussed by the team.

Key experimental controls included: Maintaining role confidentiality throughout the session; Prohibiting written notes prior to group discussion; Using identical visual stimuli (umbrella images) across conditions; Implementing strict timing protocols using computerized prompts. Conducting post-session checks for motivation manipulation efficacy.

Both tasks utilized the same prompt structure: “Umbrellas are indispensable at home! Please discuss (improvement designs/purchase plans) for the umbrella shown.” All sessions were video-recorded for behavioral coding and synchronized with fNIRS data acquisition.

### 2.3 Behavioral performance assessment

The expression of creativity on four dimensions (novelty, suitability, completeness, and refinement) was assessed on a 5-point Likert scale, with a higher score indicating higher creativity. An overall score for each group of participants on each task was obtained by calculating the average score on all dimensions. Eight graduate students from our research group were recruited to use the empathy assessment technique (Song and Shi, 2005) to evaluate the groups’ problem-solving solutions on a 5-point Likert scale (ranging from 1 = *not at all* to 5 = *very*): four of the graduate students evaluated the creative design task and the other four evaluated the purchase plan task. The average score of the four raters was taken as the final score for each group’s proposal. The inter-rater agreement was satisfactory (Cronbach’s *𝛼* = 0.837).

### 2.4 fNIRS data collection

**Figure 2:**
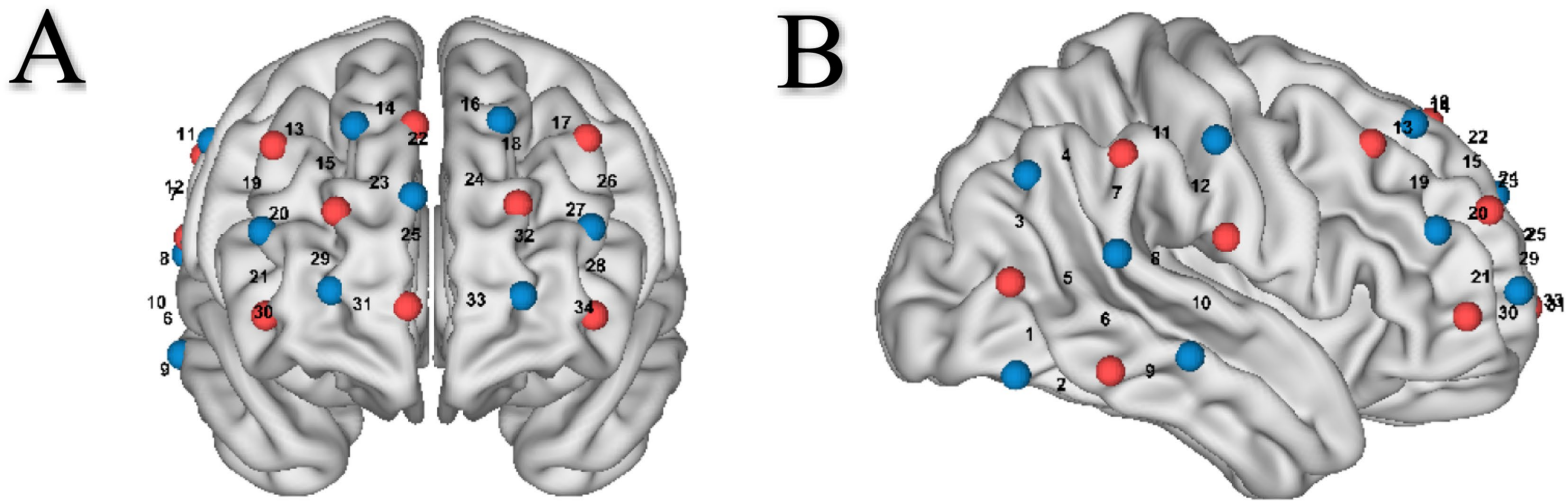
**(A)** Optode probe set on the prefrontal cortex. **(B)** Optode probe set on the right temporal parietal regions.

A NirSmart-5000A system (Danyang Huichuang Medical Equipment Co., Ltd., China) was used to continuously measure and record the concentration changes of brain oxygenated hemoglobin (HbO) and deoxyhemoglobin (HbR) during the task. The system consists of a near-infrared light source and avalanche photodiodes as detectors, with wavelengths of 730 nm and 850 nm, respectively, and a sampling rate of 11 Hz. As previous studies have indicated that the prefrontal cortex and right temporal/parietal regions are associated with creative idea generation and social interaction (Xue et al., 2018; Lu et al., 2018), we mainly focused on these brain regions. One 3 × 5 optode probe set (seven emitters and eight detectors; 3 cm optode separation) consisting of 22 measurement channels was placed over the prefrontal cortex of each participant. Meanwhile, one 3 × 3 optode probe set (five emitters and four detectors; 3 cm optode separation) consisting of 12 measurement channels was placed over the right temporal and parietal regions of each participant (Figure 2).

### 2.5 Determining the IBS increment between the corresponding channels

In previous studies, HbO signaling was more sensitive to changes in blood flow in the cerebral cortex than HbR signaling (Cui et al., 2012; Jiang et al., 2012); accordingly, we focused mainly on HbO signaling. To remove some of the global noise from the NIR signal, the NIR raw data for each individual was preprocessed in MATLAB (R2013b). Brain data from the resting and task phases were used for the subsequent brain data analysis. To obtain more stable task state data, the data collected during the 30 s before and after the task phase were eliminated. The wavelet transform coherence algorithm was then used to calculate the brain synchronization of the two individuals (Grinsted et al., 2004). We subtracted the resting state data from the task state data to obtain an indicator of the IBS increment. A Fisher *z*-transformation was performed on the IBS increments before carrying out the statistical tests (Cui et al., 2012).

**Figure 3:**
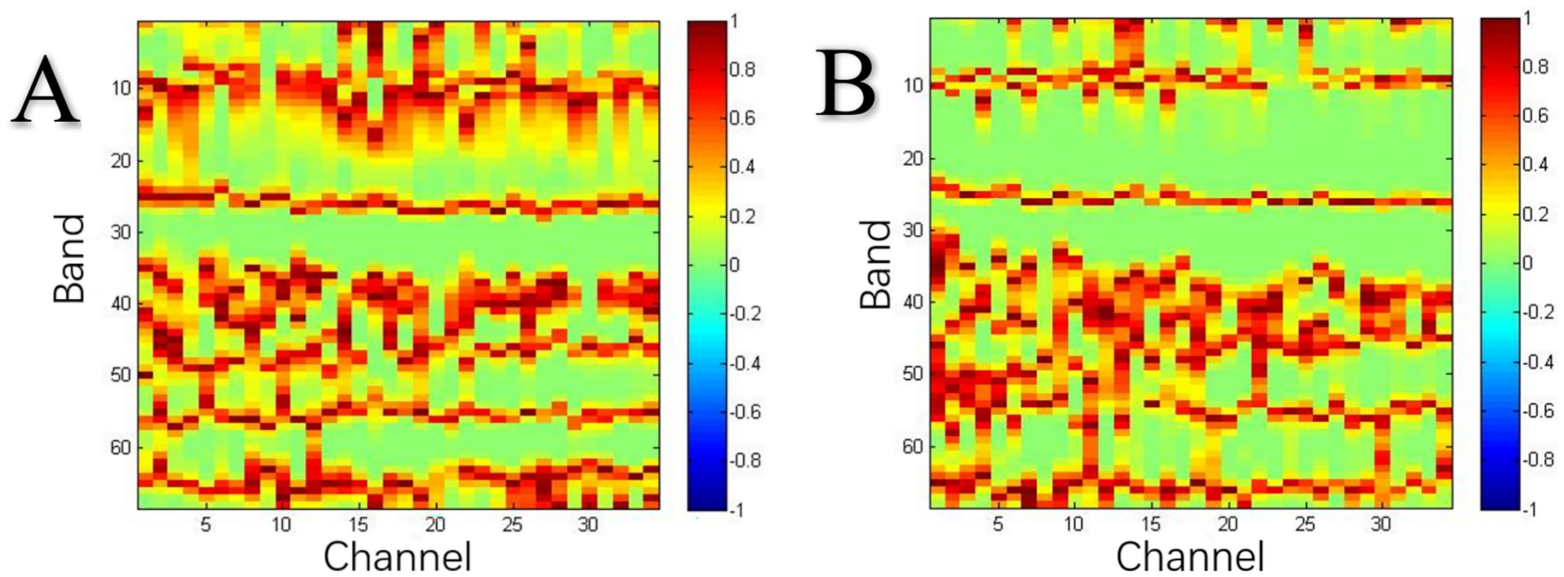
**(A)** Frequency bands of interest of the social motivation non-diversity group. **(B)** Frequency bands of interest of the social motivation diversity group.

To select the frequency bands of interest (those related to the effect of the diversity level of group social motivation on group creative activities), we performed a single-sample t-test for the IBSs (Task Stages 1 and 2) at all frequencies in the range of 0.01–0.7 Hz in the two groups, with 0 as the reference value (Nozawa et al., 2016; Pan et al., 2018). To remove some extreme low-frequency oscillation signals, we ignored frequencies below 0.01 Hz; as signals above 0.15 Hz may be affected by noises such as heartbeat activity (0.8–2.5 Hz; Barrett et al., 2015), signals above 0.15 Hz were also not considered. A threshold of *p* < 0.0005 was set for the significance of the statistical results. As the objective of this step of the analysis was only to determine the band of interest, and not to obtain the final result, no other corrections were made (Zheng et al., 2018). The band of interest was found to be in the range of 0.0762–0.1078 Hz (see Figures 3A,B).

For all subsequent analyses, we averaged the IBS of all frequencies in the band of interest, such that all reported IBS scores are the mean values within that frequency band. Again using 0 as the reference value, we performed a single-sample t-test and an independent sample t-test for the mean IBS of all (34) channels in the two groups. The false discovery rate (FDR) method was used to correct the *p*-value obtained by the independent samples *t*-test (*p* < 0.05). *Post hoc* tests were performed on significant results to correct for multiple comparisons using the Bonferroni correction method. Finally, we calculated the Pearson product difference correlation between the IBS increment and the evaluated level of group creativity on the dimensions of novelty, suitability, completeness, and refinement.

### 2.6 Pre- and post-experiment assessment

Before conducting the experiment, we measured the individual creative potential of the participants using the Runco Ideational Behavior Scale (RIBS; Runco et al., 2016). This scale showed good reliability in the current study (Cronbach’s *α* = 0.809). We also measured the personality of the individual participants using the Chinese Big Five Personality Inventory-Open Personality Subscale (CBF-OP; Dai et al., 2004), which showed good reliability (Cronbach’s *α* = 0.870), and the Cooperative and Competitive Personality Tendency Scale (CCPTS; Xie et al., 2006), which showed adequate reliability (Cronbach’s *α* = 0.732).

After the experiment was completed, we used the Cooperation Questionnaire (Shen and Benson, 2016), the Conflict Scale (Johnson et al., 2006), and the Familiarity Scale (Li, 2021) to measure the levels of cooperation, conflict, difficulty, and familiarity in the process of the task. The reliability of these scales in this study was 0.852, 0.843, and 0.692, respectively.

## 3 Results

### 3.1 Motivation activation validity test

Social motivation was tested by the independent sample *t*-test. Table 1 shows the results for the prosocial motivation and self-interested motivation groups on the social motivation scale.

**Table 1:** Results of independent sample *t*-test of social motivation (*N* = 120, df = 58).

	Motivation	*M* ± *SD*	*t*
Prosocial groups	Prosocial	9.250 ± 1.052	8.069***
	Self-interest	7.383 ± 1.451	
Self-interested groups	Prosocial	6.816 ± 2.354	−8.417***
	Self-interest	10.266 ± 2.130	

*p < 0.05, **p < 0.01, ***p < 0.001.

### 3.2 Analysis of control variables

Using social motivation group diversity as the independent variable, we performed an independent-sample t-test on the pre-experiment RIBS, CBF-OP, and CCPTS scores and on the post-experiment Cooperation Questionnaire, Conflict Scale, and Familiarity Scale scores. No significant differences were found (*p* > 0.05).

### 3.3 Behavioral performance

To examine whether there were differences in the effect of social motivation diversity on group creativity performance across the two task types, we conducted a two-factor repeated measures analysis of variance on the scores for the four dimensions of creativity performance (novelty, suitability, completeness, and refinement) with social motivation and task type as independent variables, where social motivation was an inter-subject factor and task type was an intra-subject factor.

**Figure 4:**
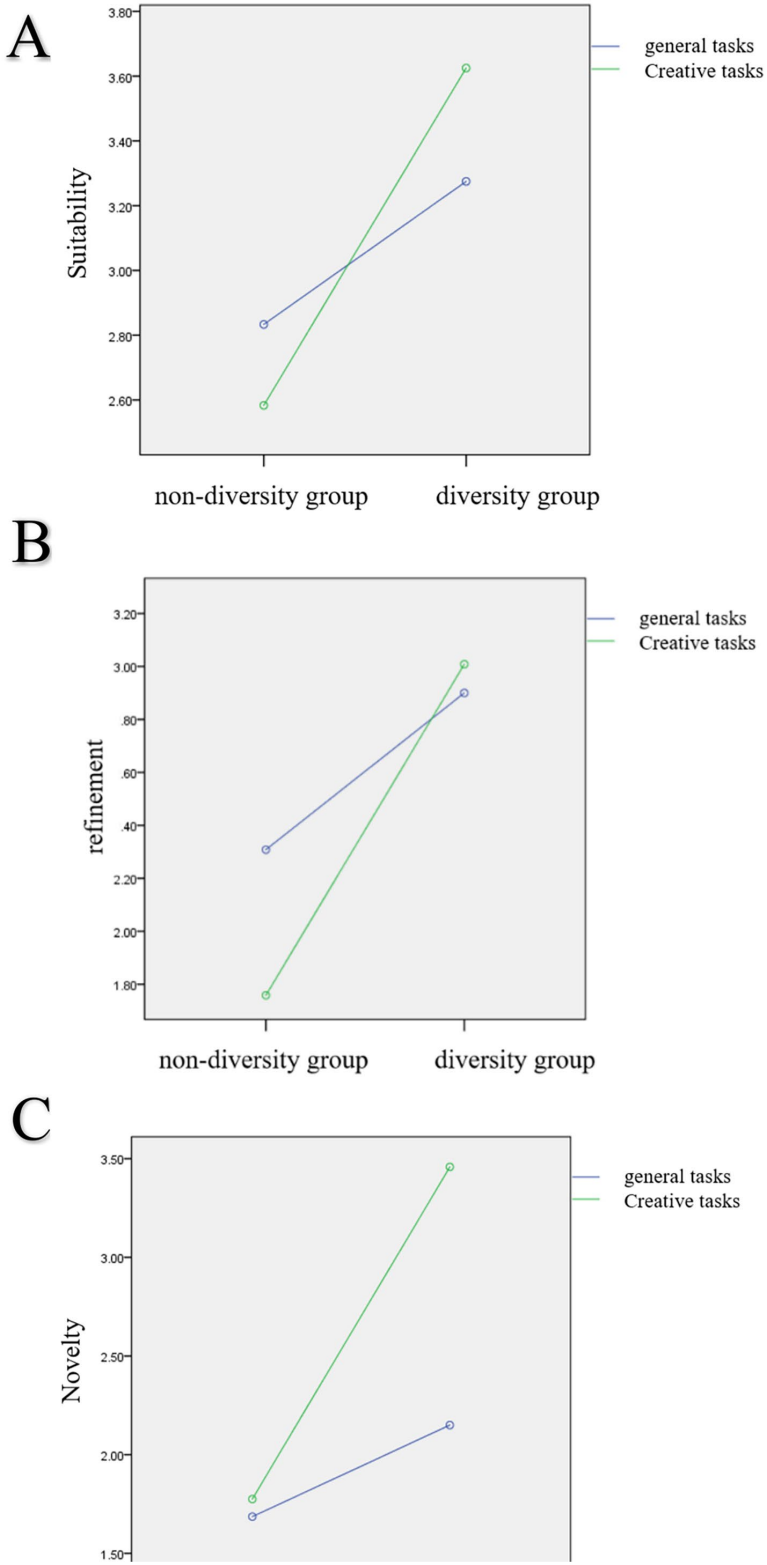
**(A)** Simple effect analysis of novelty. **(B)** Simple effect analysis of suitability. **(C)** Simple effect analysis of refinement.

On novelty, the main effect of social motivation diversity was significant, *F*(1,58) = 108.947, *p* < 0.001, partial *η*^2^ = 0.653; the main effect of task type was significant; and the interaction term social motivation diversity × task type was significant, *F*(1,58) = 58.033, *p* < 0.001, partial *η*^2^ = 0.500. A further simple effect analysis showed that the simple effect of social motivation diversity was significant for general tasks, *F* = 13.372, *p* = 0.001, partial *η*^2^ = 0.187, and for creative tasks, *F* = 158.484, *p* < 0.001, partial *η*^2^ = 0.732. The social motivation diversity group scored significantly higher in novelty than the social motivation non-diversity group in general tasks (diversity: *M* = 2.150, *SD* = 0.090; non-diversity: *M* = 1.686, *SD* = 0.090; *p* = 0.001, partial *η*^2^ = 0.187) and in creative tasks (diversity: *M* = 3.458, *SD* = 0.095; non-diversity: *M* = 1.775, SD = 0.095; *p* < 0.001, partial *η*^2^ = 0.732; see Figure 4A).

On suitability, the main effect of social motivation diversity was significant, *F*(1,58) = 42.429, *p* < 0.001, partial *η*^2^ = 0.422; the main effect of task type was non-significant; and the interaction term social motivation diversity × task type was significant, *F*(1,58) = 11.676, *p* < 0.001, partial *η*^2^ = 0.168. A further simple effect analysis showed that the simple effect of social motivation diversity was significant for general tasks, *F* = 6.974, *p* = 0.011, partial *η*^2^ = 0.107, and for creative tasks, *F* = 81.132, *p* < 0.001, partial *η*^2^ = 0.583. The social motivation diversity group scored significantly higher in suitability than the social motivation non-diversity group in general tasks (diversity: *M* = 3.275, *SD* = 0.082; non-diversity: *M* = 2.833, *SD* = 0.118; *p* = 0.011, partial *η*^2^ = 0.107) and in creative tasks (diversity: *M* = 3.625, *SD* = 0.08; non-diversity: *M* = 3.275, *SD* = 0.118; *p* < 0.001, partial *η*^2^ = 0.583; see Figure 4B).

On completeness, the main effect of social motivation diversity was significant, *F*(1,58) = 32.687, *p* < 0.001, partial *η*^2^ = 0.360; the main effect of task type was non-significant; and the interaction term social motivation diversity × task type was non-significant.

On refinement, the main effect of social motivation diversity was significant, *F*(1,58) = 43.361, *p* < 0.001, partial *η*^2^ = 0.428; the main effect of task type was significant, *F*(1,58) = 5.473, *p* < 0.05, partial *η*^2^ = 0.086; and the interaction term social motivation diversity × task type was significant, *F*(1,58) = 12.159, *p* < 0.001, partial *η*^2^ = 0.173. A further simple effect analysis showed that the simple effect of social motivation diversity was significant for general tasks, *F* = 10.140, *p* = 0.002, partial *η*^2^ = 0.149, and for creative tasks, *F* = 69.726, *p* < 0.001, partial *η*^2^ = 0.546. The social motivation diversity group scored significantly higher in refinement than the social motivation non-diversity group in general tasks (diversity: *M* = 2.900, *SD* = 0.732; non-diversity: *M* = 2.308, *SD* = 0.706, *p* = 0.002, partial *η*^2^ = 0.149) and in creative tasks (diversity: *M* = 3.008, *SD* = 0.648; non-diversity: *M* = 1.758, *SD* = 0.502; *p* < 0.001, partial *η*^2^ = 0.546; see Figure 4C).

### 3.4 Groupwise differences in IBS in the frequency bands of interest

We performed single-sample tests on the IBS of all channel combinations of the same channel in both groups during the task period (Task Stages 1 and 2), using 0 as the reference value. The obtained results were corrected using the FDR method (*p* < 0.05). The results showed no significant increase in brain synchronization on channel 2 in the social motivation non-diversity group (*p* = 0.07 > 0.05) or on channels 1 (*p* = 0.29 > 0.05), 2 (*p* = 0.28 > 0.05), and 9 (*p* = 0.06 > 0.05) in the social motivation diversity group; on the remaining channels, the IBS scores of the two groups within the frequency range of interest were significantly higher than 0.

**Figure 5:**
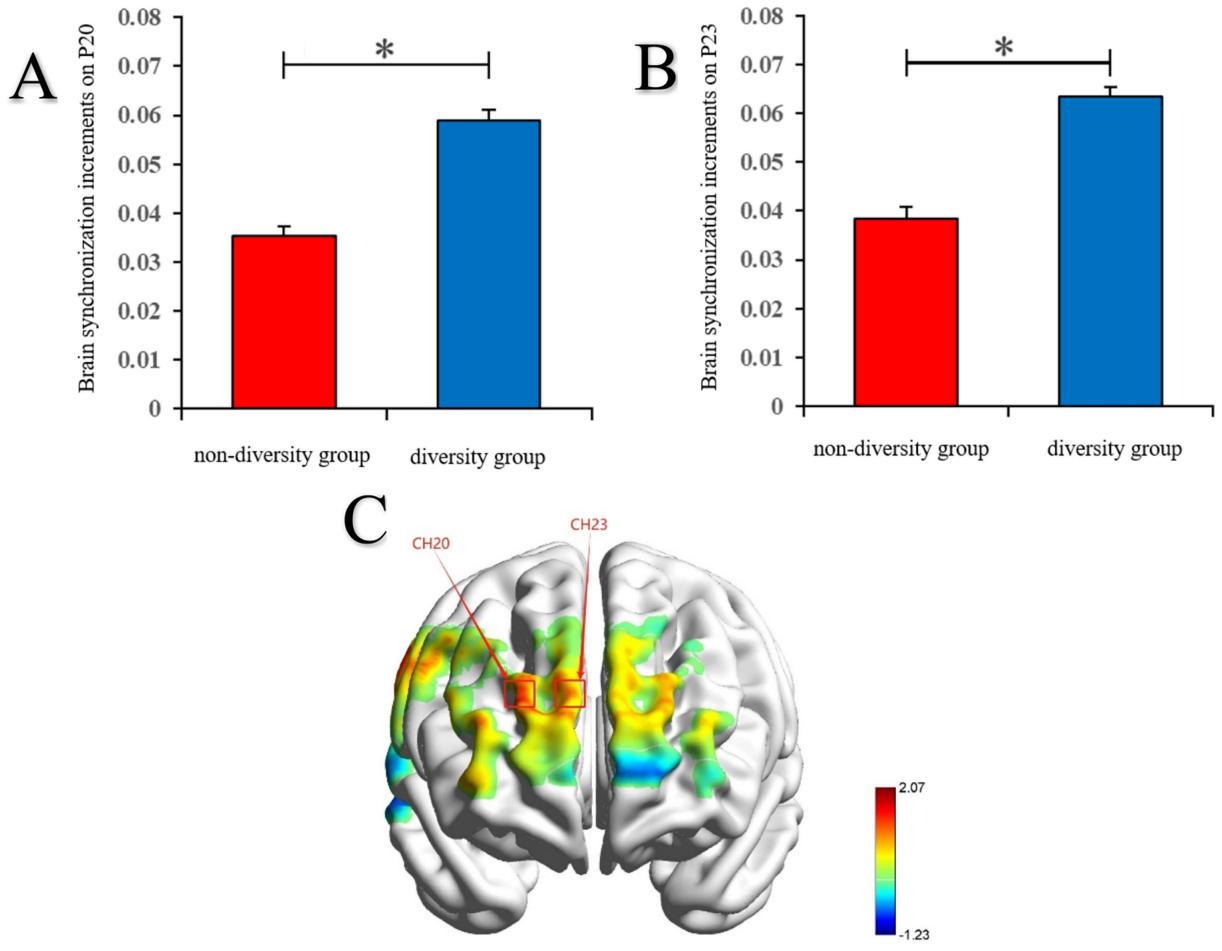
**(A)** Brain synchronization increments on CH20 in each group. **(B)** Brain synchronization increments on CH23 in each group. **(C)** Differences in brain synchronization increment between the two groups on CH20 and CH23.

With social motivation diversity as the independent variable, an independent sample *t*-test was then conducted on the IBS increments of all channel combinations of the same channel between the two groups (social motivation non-diversity group vs. social motivation diversity group) during the task period. The obtained results were corrected using the FDR method (*p* < 0.05). The results showed a significant difference in IBS increment on channel 20 between the two groups, *t*(58) = −2.0181, *p*_corr_ = 0.0482, Cohen’s *d* = 0.52998. Specifically, the IBS increment on channel 20 was significantly lower in the social motivation non-diversity group (*M* = 0.0353, *SD* = 0.0019) than in the social motivation diversity group (*M* = 0.0589, *SD* = 0.0021; see Figure 5A). There was also a significant difference in IBS increment on channel 23 between the two groups, *t*(58) = −2.0679, *p*_corr_ = 0.0431, Cohen’s *d* = 0.54306. Specifically, the IBS increment on channel 23 was significantly lower in the social motivation non-diversity group (*M* = 0.0385, *SD* = 0.0024) than in the social motivation diversity group (*M* = 0.0634, *SD* = 0.0019; see Figure 5B). The difference in IBS increments between the two groups on channels 20 and 23 is illustrated in Figure 5C.

### 3.5 IBS–behavior relationships

**Figure 6:**
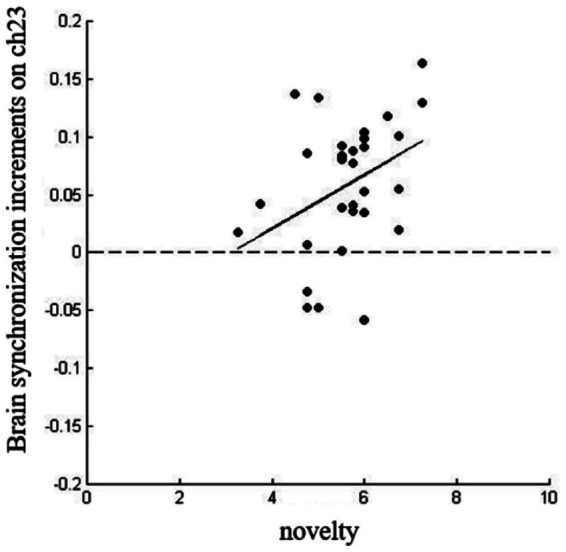
Correlation analysis for novelty and brain synchronization increment on CH23.

We conducted a Pearson product difference correlation analysis on the IBS increment and group creativity levels of the social motivation non-diversity group and the social motivation diversity group at channels 20 and 23. The results showed no significant correlation within the social motivation non-diversity group (*p*_uncorr_ > 0.05) but there was a significant correlation within the social motivation diversity group: specifically, the novelty dimension of group creativity was significantly and positively correlated with the IBS increment on channel 23 (*r* = 0.37, *p*_uncorr_ = 0.043; see Figure 6).

## 4 Conclusion

The results show that the socially motivated task exhibits a higher level of group creativity than the socially motivated non-diversity group, and there are significant differences between the two groups in the four scoring dimensions of group creativity, and the socially motivated diversity group shows higher group creativity than the socially motivated non-diversity group.

In addition, we found that the interaction between social motivation diversity and task type was significant in novelty, suitability and refinement, but not in completeness, indicating that task type can also affect the effect of social motivation diversity on group creativity.

Neuroimaging results showed that members of the socially motivated diversity group exhibited higher brain synchrony increments (IBS) between the CH20 and CH23 channels located in the prefrontal cortex than the socially motivated non-diversity group during the task.

## 5 Discussion

In this study, we divided the participants who did not know each other into a pair with diverse social motivations and a group with no diversity of social motivations. Each team is required to complete one creative task and one general task. At the same time, we used fNIRS-based ultrascan technology to record the cerebral cortical activity of two subjects in the group simultaneously and continuously. To the best of our knowledge, this is the first study that attempts to reveal the brain–brain neural basis behind the influence of group social motivational diversity on group creativity.

The results show that the socially motivated task exhibits a higher level of group creativity than the socially motivated non-diversity group, and there are significant differences between the two groups in the four scoring dimensions of group creativity, and the socially motivated diversity group shows higher group creativity than the socially motivated non-diversity group, which supports hypothesis 1. According to the theory of motivated information processing (MIP), people’s social motivation can influence the behavior of individuals in a group (De Dreu et al., 2011), and in a group, both superficial diversity (Harvey, 2013) and deep diversity (Flynn et al., 2001) are closely related to divergent thinking and creative individual and group activities. Therefore, the impact of social motivation diversity on group creativity found in this study also confirms the previous research results to a certain extent. In addition, we found that the interaction between social motivation diversity and task type was significant in novelty, suitability and refinement, but not in completeness, indicating that task type can also affect the effect of social motivation diversity on group creativity. Accordingly, hypothesis 2 is supported to some extent by this finding. This may imply that the diversity of social motivations of the group has a positive effect on the group’s level of creativity and the presentation of creative perspectives, and that this role may be influenced by the type of task (Mayseless et al., 2019).

Neuroimaging results showed that members of the socially motivated diversity group exhibited higher brain synchrony increments (IBS) between the CH20 and CH23 channels located in the prefrontal cortex than the socially motivated non-diversity group during the task, a finding that supports hypothesis 3 that IBS within the socially motivated diversity group was significantly higher than that in the socially motivated non-diversity group. Interpersonal Brain Synchronization (IBS) increases between individuals when they interact with each other, and according to the social interaction hypothesis, the increase in brain synchronization is the information transfer mechanism between the two sides of the interaction, such as the better the mutual understanding of the two interactors, the higher the brain synchronization (Hasson et al., 2012). Lu et al. (2018) explored the influence of interpersonal interaction patterns on group creative performance and the neural basis behind it, and found that group members showed an increase in IBS in the cooperative mode, which was positively correlated with creative performance, and the above-mentioned brain synchronization improvement was located in the right dorsolateral prefrontal lobe and right temporoparietal joint area. Similar to previous studies, this study also found that in groups with diverse social motivations, members of the group showed a significant increase in IBS, and the increase in IBS was significantly positively correlated with the novelty of group creativity, and this result was also found in channel 23 (CH23) located in the prefrontal lobe. In other words, there is a very close relationship between the brain synchronization and group creative activities in the above brain regions. The prefrontal cortex, as a core brain region for social cognition and creative thinking (Beaty et al., 2016), exhibits increased synchronization that reflects enhanced shared intentionality and improved efficiency in integrating perspectives among team members (Jiang et al., 2015). It is worth noting that IBS in channel CH23 (corresponding to the prefrontal cortex) was specifically associated with novelty, a finding that provides new empirical support for understanding the neural mechanisms of group innovation. However, it should be noted that while IBS is often interpreted as a marker of cognitive or affective coupling, alternative explanations exist. For example, increased synchrony could also arise from shared attention to external stimuli, similar task engagement, or non-specific physiological noise. The specificity of the association between CH23 synchrony and novelty ratings lends support to its possible functional relevance, but further research is necessary to rule out other confounding factors and establish whether IBS plays a causal role in creative collaboration. In summary, the observed IBS differences are consistent with the hypothesis that social motivation diversity enhances neural coupling in prefrontal regions, which in turn may support group creativity. Nonetheless, these interpretations remain tentative and should be evaluated in light of alternative accounts and future replications.

The practical significance of this study lies in its provision of neuroscientific evidence for constructing diverse teams in organizational management. The results indicate that in contexts requiring innovative breakthroughs, forming teams with diverse social motivations and promoting inter-brain neural coordination can effectively enhance group creativity. This discovery offers direct value for education, corporate management, and innovation training—for example, through neurofeedback techniques designed to enhance team neural synchronization or by designing collaborative tasks tailored to motivationally diverse groups.

There are several limitations to be aware of in this study. First, in this study, we only focused on the impact of the diversity of social motivations on group creativity. According to previous research, it is also possible that task types have an impact on group creativity, and this effect may need to be separated in the future. Secondly, this study found that the IBS increment on the CH20 and CH23 channels of the socially motivated diversity group was significantly higher than that of the non-diversity group of social motivation, but only the IBS increment on the CH23 channel was significantly positively correlated with the novelty of group creativity.

## Data Availability

The datasets presented in this study can be found in online repositories. The names of the repository/repositories and accession number (s) can be found: https://osf.io/28px7/?view_only=532c964811e64f39807594f019d9318d.
